# Classical and quantum conductivity in β-Ga_2_O_3_

**DOI:** 10.1038/s41598-018-38419-0

**Published:** 2019-02-04

**Authors:** David C. Look, Kevin D. Leedy

**Affiliations:** 1 Semiconductor Research Center, Wright State University, Dayton, OH 45435 USA; 2 Air Force Research Laboratory Sensors Directorate, Wright-Patterson AFB, OH 45433 USA

## Abstract

The conductivity σ, quantum-based magnetoconductivity Δσ = σ(B) − σ(0), and Hall coefficient R_H_ (= µ_H_/σ) of degenerate, homoepitaxial, (010) Si-doped β-Ga_2_O_3_, have been measured over a temperature range T = 9–320 K and magnetic field range B = 0–10 kG. With ten atoms in the unit cell, the normal-mode phonon structure of β-Ga_2_O_3_ is very complex, with optical-phonon energies ranging from kT_po_ ~ 20–100 meV. For heavily doped samples, the phonon spectrum is further modified by doping disorder. We explore the possibility of developing a single function T_po_(T) that can be incorporated into both quantum and classical scattering theory such that Δσ vs B, Δσ vs T, and µ_H_ vs T are all well fitted. Surprisingly, a relatively simple function, T_po_(T) = 1.6 × 10^3^{1 − exp[−(T + 1)/170]} K, works well for β-Ga_2_O_3_ without any additional fitting parameters. In contrast, Δσ vs T in degenerate ScN, which has only one optical phonon branch, is well fitted with a *constant* T_po_ = 550 K. These results indicate that quantum conductivity enables an understanding of classical conductivity in disordered, multi-phonon semiconductors.

## Introduction

The semiconductor Ga_2_O_3_ has five structural forms, α, β, γ, δ, and ε, the most stable of which is β-Ga_2_O_3_ (hereafter called βGAO), which crystallizes in the monoclinic form. This material has experienced extensive research activity in the last few years, mainly because of its high band gap, E_g_ = 4.6–4.9 eV, significantly higher than that of most other common wide-band-gap semiconductors, such as GaN, ZnO, and SiC^1,2^. This feature leads to a higher breakdown field^3^, important for power electronics, and also less absorption in the UV, useful for applications requiring transparency^2^. Moreover, even with this large band gap, βGAO can be highly doped with shallow donors such as Si and Sn, attaining free-electron concentrations n ≈ 2 × 10^20^ cm^−3^ ^4^. Such high concentrations enable transparent electrodes for photovoltaics and flat-panel displays, and regrown ohmic contacts^5^. Finally, homoepitaxial device technology is possible because large βGAO crystals can be grown by several different techniques^2^.

With such interesting practical applications on the horizon, it is important to understand the classical electrical properties, in particular, conductivity σ, concentration n, and Hall mobility µ_H_ = σR_H_, where R_H_ is the Hall coefficient. For binary semiconductors with only two atoms in the unit cell, such as GaN, SiC, ZnO, and ScN, the relevant scattering theory is simplified by the existence of only one branch of optical phonons. Thus, polar-optical-phonon scattering in these materials can be effectively described in terms of only one longitudinal optical phonon, of energy kT_po_^6^, where k is Boltzmann’s constant and T_po_ is the polar optical phonon temperature. (For reference, the table on p. 84 of ^6^ lists T_po_ values for sixteen binary semiconductors.) In contrast, βGAO contains ten atoms in the unit cell and thus nine branches of optical phonons, greatly complicating the analysis. The full spectrum of normal-mode phonons has recently been calculated and discussed in detail^7–10^. However, in this study, we will be concerned with *degenerate* Si-doped βGAO, which has the additional complication of disorder due to the random positions of the Si-dopant atoms^11–13^. Such disorder leads to small, negative contributions to the conductivity via a quantum effect, electron-wave constructive interference. This effect can be reduced by an increase in temperature T or magnetic-field strength B, with the latter leading to a positive magnetoconductivity (MC). We define Δσ(B,T) = σ(B,T) − σ(0,T), and will show that a theoretical analysis of Δσ as a function of B and T provides enough detail of the actual phonon spectrum to quantitatively explain µ_H_ vs T, a completely different experiment. Besides this *positive* contribution to the MC, a much more common, non-quantum, *negative* contribution to the MC can also exist^11–14^; however, it is negligible in our sample due to a high degree of degeneracy.

The film of this study was homoepitaxial, grown by pulsed laser deposition (PLD) at 550 °C on a Fe-doped (010) βGAO substrate. The substrate was semi-insulating and thus electrically isolated from the film. The growth ambient was a 5% O_2_/95% Ar gas mixture at 1.33 Pa, and the ablation target was a 99.99% pure sintered Ga_2_O_3_ disk with 1 wt. % SiO_2_. Nearly identical films have been extensively characterized by X-ray diffraction (XRD), atomic-force microscopy (AFM), and transmission electron microscopy (TEM), as presented in ref.^4^. A film thickness d = 502 nm was measured by contact profilometry. The thickness was also determined from spectral reflectance R_m_ and transmittance T_m_ measurements which can be accurately converted to the elements η and κ of the index of refraction (η + iκ) in a homoepitaxial sample^15^. At an energy E = 2 eV, η = 2.02, and Fabry-Perot oscillations (FPO) then yielded d = 508 nm, close to the profilometer value.

**Figure 1:**
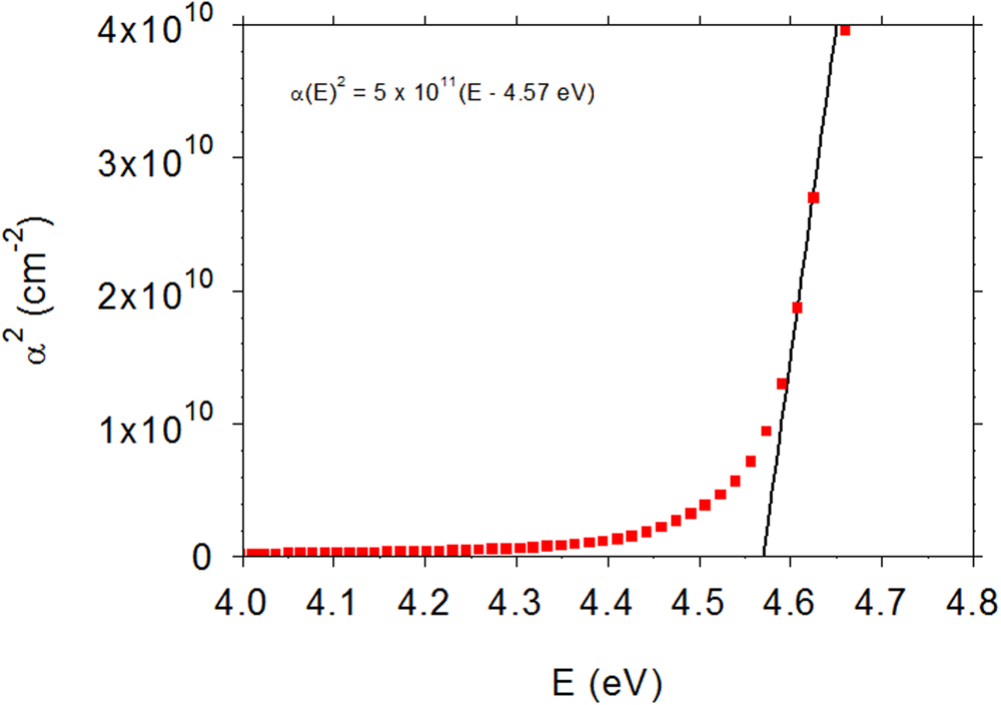
Square of absorption coefficient α vs energy E for βGAO. The intercept of α^2^ vs E gives a direct energy gap of 4.57 eV.

Another common use of R_m_ and T_m_ measurements is determination of the band gap E_g_^15^. The values of η and κ can be directly converted to absorption α and reflection R coefficients, and for crystalline materials with a direct band gap, a plot of α^2^ vs energy E will have an intercept E_g_ at E = 0. As shown in Fig. 1, the result is: α^2^ = 5 × 10^11^ (E − 4.57 eV) cm^−2^, giving E_g_ = 4.57 eV. In agreement, typical E_g_ values for βGAO mentioned in the literature are 4.5–4.9 eV^1^.

**Figure 2:**
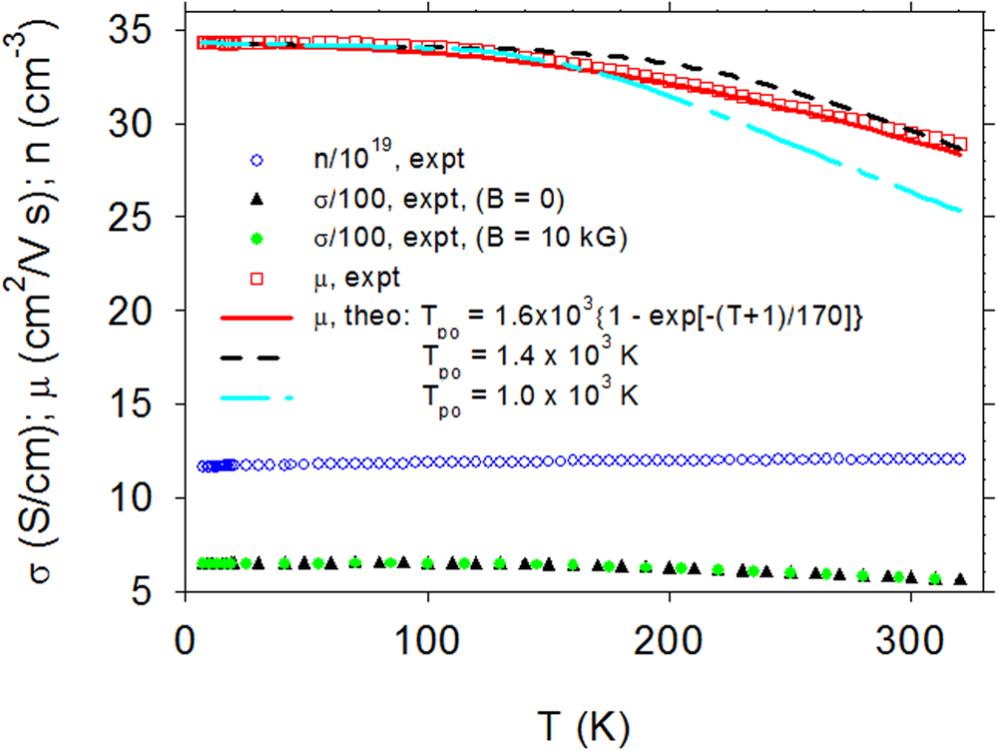
Conductivity σ, Hall-mobilty µ, and free-electron concentration n, vs temperature T in βGAO. The lines are theoretical fits of µ vs T under three possible conditions: (1) T_po_(T) = 1.4 × 10^3^ K; (2) T_po_(T) = 1.0 × 10^3^ K; or (3) T_po_(T) = 1.6 × 10^3^{1 − exp[−(T + 1)/170]} K.

**Figure 3:**
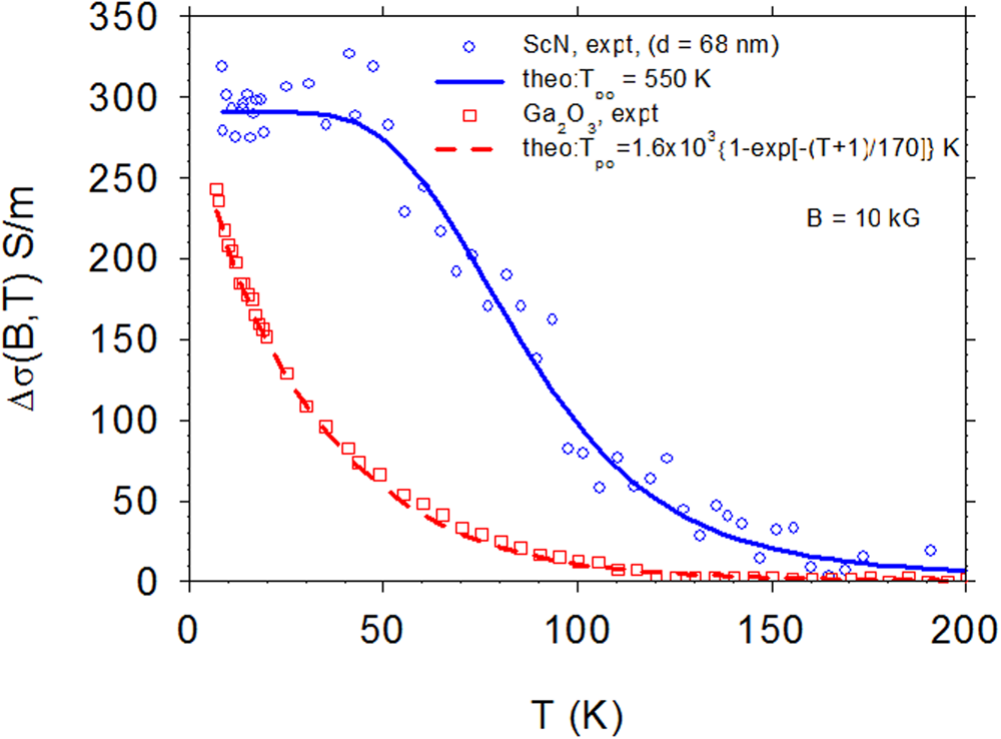
Magnetoconductivity Δσ vs T, B = 10 kG, for βGAO and thin-film ScN. The ScN is well fitted with a constant T_po_ = 550 K, but the βGAO requires a function T_po_(T) = 1.6 × 10^3^{1 − exp[−(T + 1)/170]} K.

Measurements of sheet carrier concentration n_s_, sheet conductance σ_s_, and sheet Hall coefficient R_Hs_ were carried out in a LakeShore 7507 Hall-effect system over a temperature range T = 9–320 K and a magnetic-field range, B = 0–10 kG. (All of the numbered equations in this work are in MKS units. However, in the text we will report B in “kG” rather than in the MKS unit “T” because “T” is already used for temperature. Note that 10 kG = 1 T.) For comparison with theory, σ_s_ was converted to conductivity σ = σ_s_/d, R_Hs_ to volume electron concentration n = (edR_Hs_)^−1^, and Hall mobility to µ_H_ = σ_s_R_Hs_^14^. Plots of n, µ_H_, and σ vs T are shown in Fig. 2. (Note that because n is nearly constant at 1.2 × 10^20^ cm^−3^, the layer is degenerate, and the so-called “Hall factor” is thus close to unity; in such a case, n is the true carrier concentration^14^) In Fig. 2, we have plotted σ at both B = 0 and B = 10 kG. Although the curves appear to be nearly identical on the scale of this plot, their small difference Δσ is important and is expanded and plotted vs temperature in Fig. 3.

As mentioned earlier, the βGAO unit cell has 10 atoms that generate 30 normal modes of vibration, 3 acoustic and 27 optical^7^. In this work we will be concerned with the effects of these phonons on conductivity. Acoustic phonons scatter electrons elastically, or nearly so, and can affect µ in degenerate semiconductors at low temperatures^14^. However, they will have negligible effect on electron phase and thus will not influence Δσ. Optical phonons, on the other hand, lead to inelastic scattering and will have a strong effect on Δσ^11–13^. They will also affect µ, but only at higher temperatures because temperature-independent ionized-impurity scattering, an elastic process, is much stronger than phonon scattering at low temperatures in highly-doped materials.

The random positions of the Si ions lead to a disorder that can result in a partial localization of the phonon and electron structures, known sometimes as “weak localization”^11,12^. Indeed, this disorder is the origin of the Δσ measured here. To first order, it is customary to express the altered phonon spectrum as a somewhat localized superposition of the normal modes^12^. In the spirit of that approximation, our approach here will be to find an *effective* value of T_po_ at each temperature, i.e., T_po_(T), that can correctly describe optical-phonon scattering in three independent experiments: Δσ vs T, Δσ vs B, and µ_H_ vs T.

Transport in both bulk and thin-film βGAO has been studied by several groups in the recent past^8,10,16,17^. Ma *et al*.^16^ demonstrated the critical mobility-limiting role of polar-optical phonons by analyzing µ vs T at n ~ 10^17^ cm^−3^, and also µ vs n at n ~ 10^16^–10^19^ cm^−3^ and at T ~ 77 K and 300 K. Among other things, they found that an effective value of kT_po_ ≈ 44 meV (511 K) gave a reasonable fit to a compilation (literature) of µ vs n data at 300 K. Also, in bulk, nondegenerate βGAO, Oishi *et al*. found that the high-temperature mobility is controlled by a single effective T_po_, although its value was unspecified^17^. Finally, Ghosh and Singisetti carried out a rigorous calculation of µ vs T for an ordered, nondegenerate sample with n ~ 10^17^ cm^−3^, and theory agreed well with experiment^8^. They included the effects of all the individual optical phonons and found that phonons of different energies and polarizations affected the scattering in different ways at different temperatures. For example, an optical phonon of energy ≈ 21 meV (T_po_ ≈ 244 K) dominated the mobility at 300 K^8^. At lower temperatures, other optical phonons became important and of course it was also necessary to add the scattering contributions of acoustic phonons and ionized impurities. In another theoretical work, Kang *et al*.^10^ performed first-principles calculations on the electron and phonon structures and also calculated scattering rates and mobilities. In agreement with the conclusions of Ghosh and Singisetti^8^, they showed that many phonons contribute to the scattering. Moreover, they pointed out the dominance of polar vs nonpolar optical scattering and showed that, contrary to other assertions, the mobility does not have a large anisotropy. In principal, detailed and rigorous calculations such as those described above could be carried out for all lightly-doped, ordered βGAO samples; however, the disorder arising from heavily-doped samples will modify the actual phonon spectrum and require a more complicated analysis^12^.

We first consider the classical theory of µ vs T in degenerate semiconductors, standard in the literature^18^ except for the treatment of optical-phonon scattering. (In the equations below, the effective mass m*, static dielectric constant ε_0_, and high-frequency dielectric constant ε_1_ were taken from ref.^7^ and the acoustic deformation constant E_1_ and longitudinal elastic constant c_l_ from ref.^16^, noting that c_l_ = ρ_dens_s^2^, where ρ_dens_ is the mass density and s is the speed of sound. The only fitted parameter in our study is T_po_.) For degenerate materials, the dominant scattering mechanisms are typically ionized impurities (“ii”), acoustic phonons (“ac”), and optical phonons (“po”). The existence of degeneracy greatly simplifies the calculations, because all scattering basically occurs at one energy, the Fermi energy, E_F_ = (ħ^2^/2 m*)(3π^2^n)^2/3^, where ħ is the reduced Planck’s constant. For degenerate electrons, Matthiessen’s Rule^14,18^ applies exactly:

1 $$\mu (n,{N}_{D},{N}_{A},T)={[{\mu }_{ii}{(n,{N}_{D},{N}_{A})}^{-1}+{\mu }_{ac}{(n,T)}^{-1}+{\mu }_{po}{(n,T)}^{-1}]}^{-1}$$

We will assume that the dominant donor has charge Z_D_, and the acceptor, Z_A_; then n = Z_D_N_D_ − Z_A_N_A_^18^. In our case, the dominant donor is the dopant Si_Ga_, with Z_D_ = 1. The form of Eq. 1 assumes that a relaxation time τ can be defined for each scattering mechanism, i.e., µ = eτ/m*. This criterion holds for elastic scattering (ii and ac) but not necessarily for inelastic scattering (po), discussed further below. From the degenerate Brooks-Herring ionized-impurity scattering theory^14^, µ_ii_ can be written

2 $${\mu }_{ii}(n)=\frac{24{\pi }^{3}{{\varepsilon }_{0}}^{2}{\hslash }^{3}}{{e}^{3}{({m}^{\ast })}^{2}}\frac{1}{\mathrm{ln}(1+y(n))-\frac{y(n)}{1+y(n)}}\frac{n}{{Z}^{2}{N}_{ii}}$$

where Z^2^N_ii_ ≡ N_ii,eff_ = Z_D_^2^N_D_ + Z_A_^2^N_A_, where N_ii,eff_ is the *effective* concentration of ionized impurities^18^. [Note that N_ii,eff_ is the only fitted parameter in Eq. 2, and from it we can calculate N_D_ and N_A_: N_D_ = (N_ii,eff_ + nZ_A_)/(1 + Z_A_) and N_A_ = (N_ii,eff_ − n)/ Z_A_ (1 + Z_A_).] In Eq. 2, y(n) can be written^14^

3 $$y(n)=\frac{{3}^{1/3}4{\pi }^{8/3}{\varepsilon }_{0}{\hslash }^{2}{n}^{1/3}}{{e}^{2}{m}^{\ast }}$$

The other two scattering terms in Eq. 1 are:

4 $${\mu }_{ac}(n,T)=\frac{\pi {\hslash }^{4}{c}_{l}}{{2}^{1/2}{({m}^{\ast })}^{5/2}{{E}_{1}}^{2}ekT}{E}_{f}{(n)}^{-1/2}=\frac{{\pi }^{1/3}{\hslash }^{3}{c}_{l}}{{3}^{1/3}{{m}^{\ast }}^{2}{{E}_{1}}^{2}ekT{n}^{1/3}}$$

5 $${\mu }_{po}(T)=\frac{4\pi \varepsilon {}_{0}(3/\pi {)}^{1/3}{\hslash }^{3}{n}^{1/3}T{\sinh }^{2}(\frac{{T}_{po}}{2T})}{ek{{T}_{po}}^{2}{({m}^{\ast })}^{2}({\varepsilon }_{0}/{\varepsilon }_{1}-1)}$$

Equation 4 is well-known^18^, but Eq. 5 is not in the literature, to our knowledge. To derive Eq. 5, we begin with Eq. 17 in the theoretical paper^19^ of Howarth and Sondheimer (HS). These authors use a variational theory to show that even for polar optical phonon scattering a relaxation time τ(E) can be defined as long as T > T_po_. This is an important result, but HS-Eq. 17 can be made more useful by including the concept of an effective charge, introduced earlier for materials that have partially-ionic/partially-covalent bonds^20,21^. We make use of the Callen effective charge e_c_^20^, which can be written as e_c_^2^ = (kT_po_/ħ)^2^(ε_0_/ε_1_ − 1)M_r_a^3^, where M_r_ is the reduced ion mass and a^3^ is the volume of the unit cell. (It turns out that both M_r_ and a^3^ cancel out in the final equation.) Besides substituting e_c_^2^ for e^2^ we multiply HS-Eq. 17 by 4πε_0_ for conversion to MKS units.

For degenerate electrons, in which case scattering occurs only at the Fermi energy, Eq. 5 gives τ ∝ n^1/3^, a relationship also noted in earlier work^22^. For lower temperatures, i.e., T < T_po_, Eq. 5 must be corrected according to a table provided by HS^19^. For our calculated values of T_po_/T, shown below to range from about 5–10, the corrections are less than about 20%. Therefore, we will assume the approximate validity of Eq. 5 in order to maintain simplicity and convenience. This assumption is partially justified by the fact that Eqns. 2–5 fit the experimental mobility data quite well, as shown in Fig. 2.

We next consider the quantum-based magnetoconductivity, Δσ(B,T). Conductivity in disordered materials is affected by the wave nature of the electrons as they diffuse and scatter from point to point. This process is perhaps best understood in terms of Feynman’s approach to quantum mechanics, the path integral method. An electron diffusing from point A to point B may have many potential paths, all of which must be included in a sum of amplitudes. Even if coherence is maintained, the phases in general will not add constructively because the travel distances will vary. However, there are some paths, those containing loops, for which a portion of the travel can generate constructive interference. That is, one path can have the electron traversing in one direction around the loop, and a second path, in the other direction. Since exactly the same travel distance will occur for each, they will interfere constructively and thus be more probable than a similar path without a loop. Therefore, paths with loops are favored, and they will generate a certain amount of “backscattering” of the electron, which will reduce conductance. (This effect is not large; for our sample Δσ/σ ≈ 0.004 at T = 9 K and B = 10 kG.) All of the discussion so far assumes that coherence is maintained during the loop travel, which is true if all of the scattering is elastic. However, as temperature is increased, inelastic scattering from the optical phonons will begin to affect the electron energy and randomize its phase. Also, the presence of a magnetic field will affect phase. Both of these effects are quantified in the 3D perturbation theory of Kawabata^13^:

6 $${\rm{\Delta }}\sigma (B,T)=\frac{{e}^{2}}{2{\pi }^{2}\hslash l(B)}\sum _{N=0}^{{\rm{\infty }}}2[{(N+1+\delta (B,T))}^{1/2}-{(N+\delta (B,T))}^{1/2}]-\frac{1}{{(N+\frac{1}{2}+\delta (B,T))}^{1/2}}$$

where e^2^/2π^2^ħ = 1.23 × 10^−5^ S, is the so-called unit of quantum conductance. Also, l(B) = (ħ/eB)^1/2^ and

7 $$\delta (B,T)=\frac{{l}^{2}(B)}{4{\tau }_{po}(T)D(T)}=\frac{3e}{4\hslash {(3{\pi }^{2}n)}^{2/3}{\mu }_{po}(T)\mu (T)B}$$

where τ_po_ is the inelastic-scattering relaxation time and D(T) is the diffusion coefficient. We have modified Eq. 7 by setting τ_po_ = m*µ_po_/e and D = v_F_^2^τ/3 = ħ^2^(3π^2^n)^2/3^µ/3em*, where µ_po_ is given by Eq. 5 and n and µ are measured quantities. A remarkable consequence of Eq. 6 occurs in the limit of low T (which gives high µ_po_) and large B which renders δ ≪ 1 and

8 $${\rm{\Delta }}\sigma (B,T)=\frac{0.605{e}^{2}}{2{\pi }^{2}\hslash l(B)}=\frac{0.605{e}^{5/2}}{2{\pi }^{2}{\hslash }^{3/2}}{B}^{1/2}=290.8{B}^{1/2}S/m$$

This result is independent of temperature or any material parameter!

**Figure 4:**
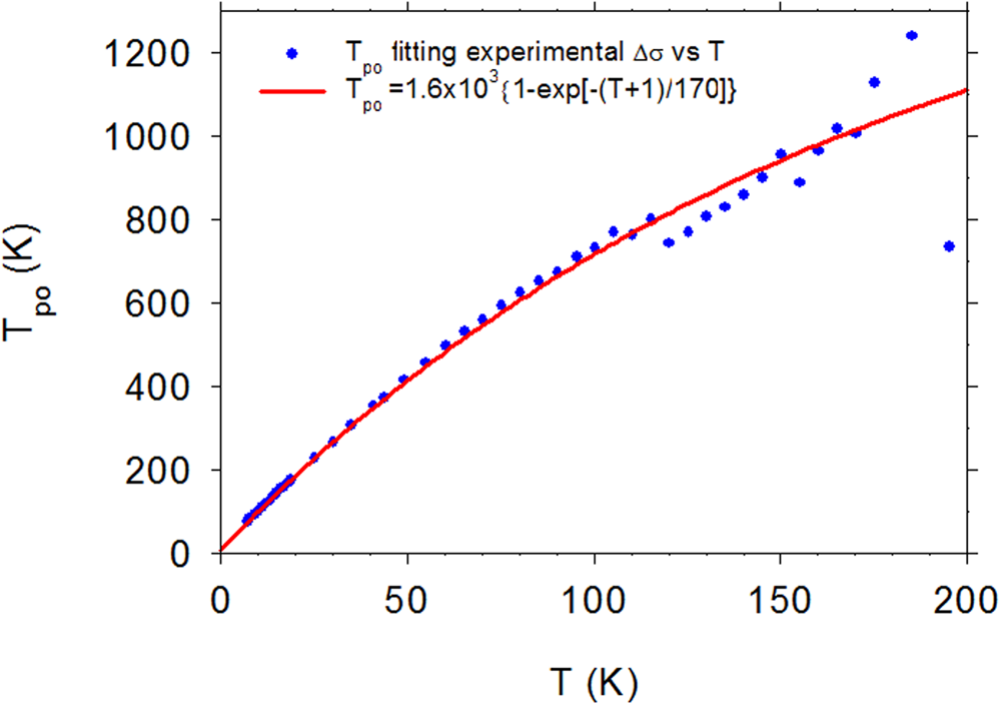
Experimental points: the required value of T_po_ to fit Δσ vs T, B = 10 kG, at *each* temperature T, for βGAO. Solid line: a fit of T_po_ vs T, giving T_po_(T) = 1.6 × 10^3^{1 − exp[−(T + 1)/170]} K.

**Figure 5:**
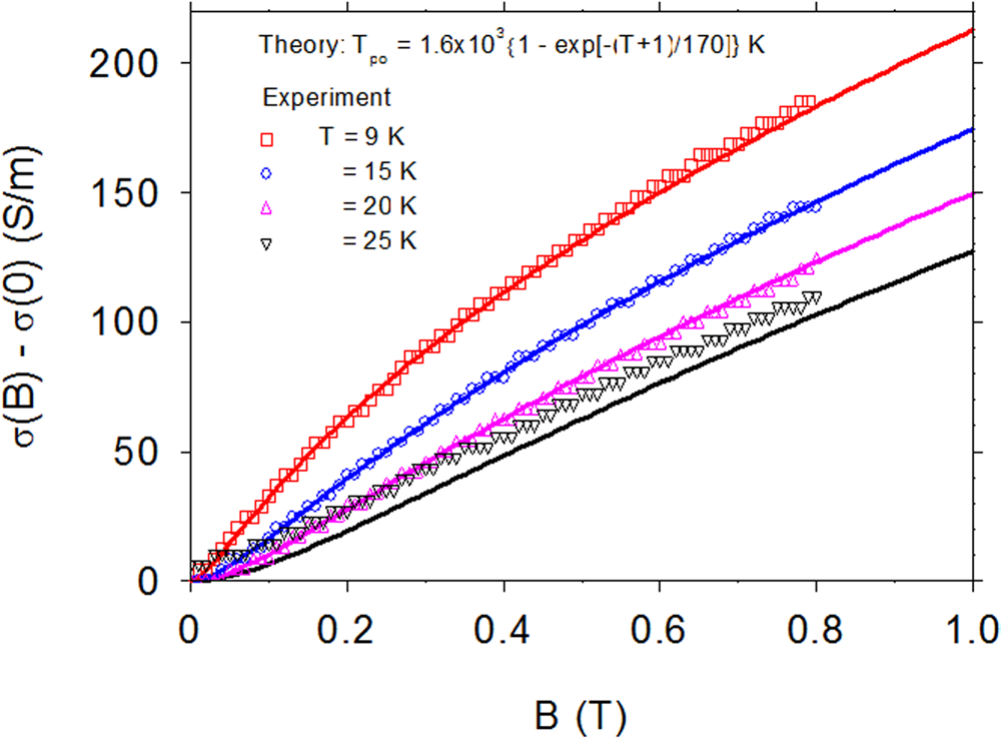
Experimental points: magnetoconductivty Δσ = σ(B) - σ(0) at temperatures T = 9, 15, 20, and 25 K. Solid lines: theoretical fits with T_po_(T) = 1.6 × 10^3^{1 − exp[−(T + 1)/170]} K.

Equation 6 is applied to experimental results for βGAO in Fig. 3, which displays Δσ vs T at B = 10 kG. It is instructive to compare the same function in thin-film, degenerate ScN. Note that ScN has only two atoms per unit cell and thus only one optical branch, which can be represented by a single value of T_po_. As seen in Fig. 3, the value T_po_ = 550 K fits the ScN data very well. (A more detailed study of magnetoconductance in ScN will be presented elsewhere.) However, a single T_po_ is not sufficient for βGAO, and indeed, we find the required T_po_, at a given T, by solving Eq. 6 as a transcendental equation with T_po_ as the unknown. The resulting points T_po_ vs T are plotted in Fig. 4, and they can be reasonably well fitted by the relatively simple function T_po_(T) = 1.6 × 10^3^{1 − exp[−(T + 1)/170]} K, shown as a solid line. The validity of this equation can then be further tested via an independent experiment, Δσ(B, T) = σ(B, T) − σ(0,T) vs B, B = 0–8 kG, T = 9, 15, 20, and 25 K. (For this particular experiment, 8 kG was the maximum field that could be used.) As seen in Fig. 5, the fit is excellent for T = 9, 15, and 20 K, and acceptable for T = 25 K, where the signal is rapidly decreasing due to inelastic scattering.

The final test of T_po_(T) is its applicability in a third independent experiment, µ_H_ vs T, illustrated in Fig. 2. Here we apply Eqns 1–5, comparing three choices of T_po_ in Eq. 5: 1000 K, 1400 K, or our derived function T_po_(T). The first two values were chosen to bracket the potential fits attained with T_po_ = constant; however, neither is satisfactory, nor is any other constant value. On the other hand, the function T_po_(T) works very well. We are now left with only one unknown in Eqns 1–5, N_ii,eff_, which turns out to be 5.97 × 10^20^ cm^−3^ from the solid-line fit shown in Fig. 2. We know that the dominant donor concentration N_D_ = [Si_Ga_], and we can speculate that the dominant acceptor is the Ga vacancy, V_Ga_^23,24^. The latter, if isolated, would have a charge Z_A_ = 3, but if complexed with Si, Z_A_ could be 2, or even 1. As shown earlier, we can then calculate N_D_ and N_A_ from N_ii,eff_. Under the assumptions, Z_D_ = 1 and Z_A_ = 1, 2, or 3, the results are: N_D_ = 3.57, 2.77, or 2.37 × 10^20^ cm^−3^; and N_A_ = 2.40, 0.80, or 0.40 × 10^20^ cm^−3^, respectively. To decide among these three possibilities it would be helpful to determine [Si] from another source, such as secondary ion mass spectroscopy, and [V_Ga_] from positron annihilation or electron paramagnetic resonance.

In summary, we have used the quantum-based magnetoconductivity Δσ vs T to develop a function T_po_(T) that quantitatively explains not only Δσ vs T but also Δσ vs B and µ_H_ vs T in degenerate βGa_2_O_3_. We also showed that the behavior of σ, Δσ, and µ_H_ in βGa_2_O_3_ is much different than that in ScN, a simpler system for which a *constant* T_po_ well explains both Δσ vs T and µ_H_ vs T. The methodology used to develop the function T_po_(T) is directly applicable to other complex semiconductors.

## References

[1] Higashiwaki M, Jessen GH (2018). Guest Editorial: The dawn of gallium oxide microelectronics. Appl. Phys. Lett..

[2] Baldini M, Galazka Z, Wagner G (2018). Recent progress in the growth of β-Ga_2_O_3_ for power electronics applications. Mater. Sci. in Semiconductor Processing.

[3] Green A (2016). 3.8-MV/cm breakdown strength of MOVPE-grown Sn-doped β-Ga_2_O_3_ MOSFETs. IEEE Electron Device Letters.

[4] Leedy KD (2017). Highly conductive homoepitaxial Si-doped Ga_2_O_3_ films on (010) β-Ga_2_O_3_ by pulsed laser deposition. Appl. Phys. Lett..

[5] Xia Z (2018). Delta Doped β-Ga_2_O_3_ Field Effect Transistors With Regrown Ohmic Contacts. IEEE Electron Device Letters.

[6] Rode, D. L. Low-field Electron Transport. *Semiconductors and Semimetals* **10**, 1–89, (cf. Table on p. 84.) (1975).

[7] Schubert M (2016). Anisotropy, phonon modes, and free charge carrier parameters in monoclinic β-gallium oxide single crystals. Phys. Rev. B.

[8] Ghosh K, Singisetti U (2016). *Ab initio* calculation of electron-phonon coupling in monoclinic β-Ga_2_O_3_ crystal. Appl. Phys. Lett..

[9] Sturm C, Schmidt-Grund R, Zviagin V, Grundmann M (2017). Temperature dependence of the dielectric tensor of monoclinic Ga_2_O_3_ single crystals in the spectral range 1.0−8.5 eV. Appl. Phys. Lett..

[10] Kang Y, Krishnaswamy YK, Peelaers H, Van de Walle CG (2017). Fundamental limits on the electron mobility of β-Ga_2_O_3_. J. Phys. Condens. Matter.

[11] Lee PA, Ramakrishnan TV (1985). Disordered electronic systems. Rev. Modern Phys..

[12] Dugdale, J. S. *The Electrical Properties of Disordered Metals* (Cambridge University Press, Cambridge, 2005).

[13] Kawabata A (1980). Theory of Negative Magnetoresistance in Three-Dimensional Systems. Solid State Commun..

[14] Look, D. C. Electrical Characterization of GaAs Materials and Devices (Wiley, New York, 1989).

[15] Look DC, Wang B, Leedy KD (2017). Model-free determination of optical constants: application to undoped and Ga-doped ZnO. Opt. Eng..

[16] Ma N (2016). Intrinsic electron mobility limits in β-Ga_2_O_3_. Appl. Phys. Lett..

[17] Oishi T, Koga Y, Harada K, Kasu M (2015). High-mobility β-Ga_2_O_3_(-201) single crystals grown by edge-defined film-fed growth method and their Schottky barrier diodes with Ni contact. Appl. Phys. Express.

[18] Look DC (2011). Self-compensation in semiconductors: The Zn vacancy in Ga-doped ZnO. Phys. Rev. B.

[19] Howarth DJ, Sondheimer EH (1953). The theory of electronic conduction in polar semiconductors. Proc. Roy. Soc. A.

[20] Callen HB (1949). Electric Breakdown in IonicCrystals. Phys. Rev..

[21] Böer, K.W. *Survey of Semiconductor Physics* (Van Nostrand Reinhold, New York, 1990).

[22] Zawadzki W, Szymanska W (1971). Phys. Stat. Solidi B.

[23] Korhonen E (2015). Electrical compensation by Ga vacancies in Ga_2_O_3_ thin films. Appl. Phys. Lett..

[24] Kananen BE, Halliburton LE, Stevens KT, Foundos GK, Giles NC (2017). Gallium vacancies in β-Ga_2_O_3_ crystals. Appl. Phys. Lett..

